# Examining management and research priorities in patients with polymyalgia rheumatica: a primary care questionnaire survey

**DOI:** 10.1007/s10067-018-04405-0

**Published:** 2019-01-07

**Authors:** Chris Morton, Sara Muller, Milica Bucknall, Kate Gilbert, Christian D. Mallen, Samantha L. Hider

**Affiliations:** 1 Research Institute for Primary Care and Health Sciences, Keele University, Keele, UK; 2 PMRGCAuk, London, UK; 3 Haywood Academic Rheumatology Centre, Midlands Partnership Foundation Trust, Stafford, UK

**Keywords:** Patient involvement, Polymyalgia rheumatica, Research priorities, Surveys and questionnaires

## Abstract

**Introduction/objectives:**

Polymyalgia rheumatica (PMR) is a common inflammatory disorder that is usually managed with oral glucocorticoids, which although effective can cause significant adverse events. Support group survey data suggests length of glucocorticoid treatment and managing side effects are key priority areas of management for patients. Recognising that not all patients will access patient support organisations, our objective was to identify priorities for PMR management and research among primary care PMR patients.

**Method:**

All adults aged ≥ 50 years registered with 150 English general practices who had a first read code for PMR in their medical records in the preceding 3 years were mailed a self-completion questionnaire (*n* = 704). Survey items included questions regarding patient priorities for PMR management (from a pre-defined list of 10 items) and suggestions for future research (8 items, plus a free-text option), which were developed in collaboration with PMRGCAuk.

**Results:**

Five hundred fifty patients responded (78%). The mean (SD) age was 74.1 (8.5) years and 361 (66%) were female. Priority research areas were focused on how to better manage pain, stiffness and fatigue (431, 78%), improving the diagnosis of PMR (393, 71%) and steroid management (342, 62%).

**Conclusions:**

This survey of PMR patients suggests that symptom management, early diagnosis and managing medication are key areas for patients for future research. Researchers and funding organisations should be aware of these priorities if we are to generate research findings that are relevant to the widest range of stakeholders.

## Introduction

Polymyalgia rheumatica (PMR) is an inflammatory disorder of older adults with a lifetime prevalence of 2.4% in women and 1.7% in men [1]. It classically causes pain and stiffness in the shoulders and hip girdles, which can lead to significant levels of physical disability [2]. The mainstay of treatment is oral glucocorticoids, which whilst effective are often required for prolonged periods [3]. This places patients at potential risk of adverse events and is a key concern for both patients [4] and clinicians [5].

There is increasing evidence that there is a mismatch between what research patients want to see undertaken and research being performed [6]. To address this disparity, the James Lind Alliance Priority Setting Partnerships were created, where partnerships of patients, carers and health professionals discussed and agreed on important priorities for treatment and research in a range of health conditions, such as type 1 diabetes and stroke. An evaluation of these partnerships [6] suggested that drug trials were preferred by researchers, and non-drug treatments are preferred by patients, carers and clinicians.

Involving patients in research is both best practice [7] and increasingly becoming key to securing research funding with many major funding bodies. However, reviews suggest that although patients should play an active role in setting research priorities, such participation remains the exception rather than the rule [8]. The charity PMRGCAuk was established in 2010 as an online community of patients with PMR or giant cell arteritis. The charity had previously surveyed its membership [9] to identify key priorities for PMR patients for information and support together with areas they wished to see prioritised for future research. Responses suggested that managing glucocorticoids was the key priority for the majority of respondents [9].

Patients who choose to join or access patient support groups or charities may be different to the wider population with a specific condition, with data from cancer survivors suggesting that those accessing support groups were likely to be female [10–12], younger [11, 13] and of Caucasian ethnicity [10, 12]. To investigate the broadest range of patient experience, we sought to survey a primary care population of people with PMR, using similar questions to those identified as important in the survey by PMRGCAuk [9]. Therefore, the aim of this study was to examine patient priorities for living with PMR and their priorities for PMR research within a primary care population.

## Materials and methods

### Study design and population

A cross-sectional questionnaire study was developed to investigate the impact of PMR. Adults (age ≥ 50 years) with a first read-coded diagnosis of PMR between January 1, 2010 and January 1, 2013 were identified via an electronic search of primary care records from 150 participating general practices across England. The research lead from each practice screened the list of identified patients and removed those in potentially vulnerable groups (e.g. those with significant cognitive impairment or a terminal diagnosis). Those eligible to participate (*n* = 704) were mailed a study pack, including a questionnaire and consent to participate; non-responders were sent a reminder postcard at 2 weeks and a repeat study pack at 4 weeks. Ethical approval for the study was obtained from NRES-West Midlands-Staffordshire (Ref 13/WM/0133).

Primary care records were used to establish disease duration, taken as time from date of diagnosis to date of questionnaire response. Other results in this article are derived from the questionnaire data. This questionnaire included items relating to sociodemographics (age, gender and personal circumstances), PMR characteristics (e.g. whether currently experiencing symptoms) and health information-seeking behaviour (e.g. whether a doctor had provided written information on PMR). Following collaborative work with the charity PMRGCAuk, we used two questionnaire items related to priorities around PMR which the charity had identified from surveys of users of their telephone helpline [9]. The first item presented ten aspects of living with PMR (e.g. managing pain, see Table 2); participants were asked to select five as priorities. Specifically, the question was “What in your opinion are the most important aspects of living with PMR that people need help with? This help might be information or support?” The second item presented nine areas for further PMR research (e.g. diagnosis, see Table 3): participants were asked to select any number of these as priorities.

### Data analysis

Responders and non-responders to the questionnaire were compared in terms of age and gender using a *t* test (equal variances assumed) and a chi-squared test respectively, to check for evidence of response bias. Other statistics calculated were descriptive: the count and percentage of participants that had selected each aspect of living with PMR or research area as a priority were recorded.

The second questionnaire item regarding research priorities included a free-text ‘other’ option. Responses to the ‘other’ option were categorised using content analysis. Content analysis is a systematic method for interpreting meaning in textual data [14]. First, a single author (CM) read the free-text responses repeatedly in order to gain familiarity with them as a whole. The words capturing the key concept in each response were highlighted. During this process, codes emerged that reflected the key concept in multiple responses. For example, “Why PMR develops and ways to prevent it” and “Causal factors - I blame mine on gall bladder removal” were both coded as “Causes of PMR”. The process was iterative; at each stage, responses could be recoded and codes could be relabelled, created or removed, until all responses were coded to the author’s satisfaction.

A second author (SM) independently coded a random sample of 20 responses using the codes identified by CM. The two authors then compared and discussed their choices. Although there was disagreement on the coding of only one response, the purpose of this exercise was not calculating a statistical rate of agreement. Instead, the emphasis was on bringing the authors’ different perspectives to bear on data interpretation. Disagreement or uncertainty regarding the coding of individual responses was resolved by consensus to establish final categories (Table 4). Responses that were illegible were coded as “do not know” or which had no clear connection to research priorities were removed at this stage.

## Results

**Fig. 1:**
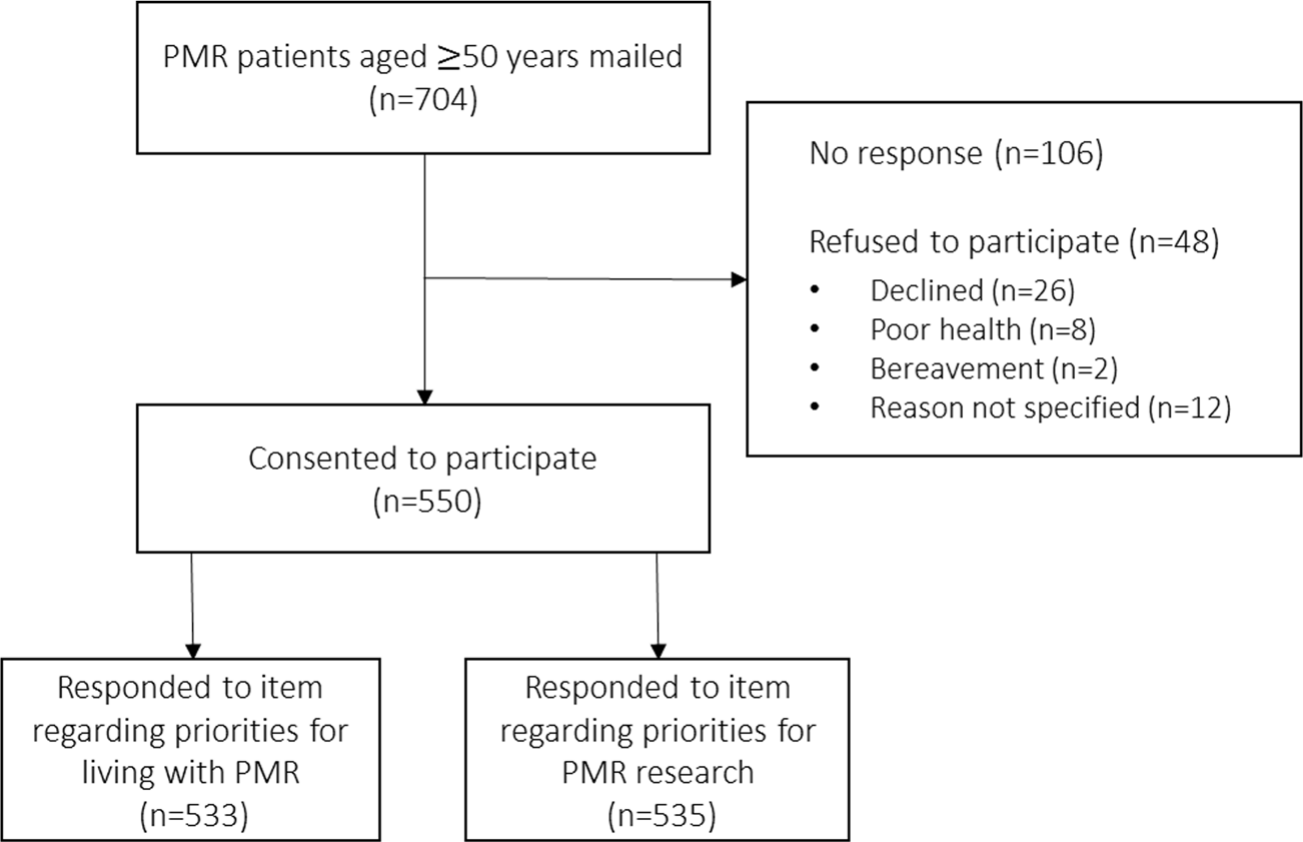
Study flow diagram

Of the 704 patients that were mailed a questionnaire, 550 (78%) consented to participate (Fig. 1). Non-responders and refusals were older than participants (mean (SD) 75.2 (9.2) years versus 74.1 (SD 8.5), *p* = 0.17) and more often female (*n* = 112 (73%) versus *n* = 361 (66%), *p* = 0.14), although these differences were not statistically significant. Consistent with other PMR studies, the majority of the sample was female (*n* = 361, 66%), with a mean age of 74.1 (SD 8.5) years (Table 1). Median (IQR) time since diagnosis was 2.0 (1.3, 2.6) years. Sixty-eight percent (*n* = 374) of participants were still experiencing PMR symptoms at the date of response. Access to PMR-related information was mixed; 50% of participants (*n* = 273) reported receiving written information whilst 43% (*n* = 234) had used the Internet to research PMR. Only 10 participants (2%) had contacted a patient support group.

**Table 1:** Characteristics of *n* = 550 participants

Variable		*N* (%)^a^
Sociodemographic		
Gender	Female	361 (66)
Age	Mean (SD), 74.1 (8.5)	
Employment status	Employed	50 (9)
	Retired	446 (81)
	Unemployed/seeking work	2 (< 1)
	Not working due to ill health	8 (1)
	Housewife/husband	29 (5)
	Other	12 (2)
Ethnicity	White	539 (99)
	Other	7 (1)
PMR characteristics		
Current PMR symptoms	Yes	374 (68)
	No	152 (28)
Years since diagnosis	Median (IQR), 2.0 (1.3, 2.6)	
Health information-seeking behaviour		
Received written information about PMR from their doctor	Yes	273 (50)
	No	261 (47)
Has internet access	Yes	302 (55)
	No	233 (42)
Used the internet to research PMR	Yes	234 (43)
	No	294 (53)
Contacted a patient support group	Yes	10 (2)
	No	527 (96)
Needs help reading documents from their doctor or pharmacy	Always	15 (3)
	Often	17 (3)
	Sometimes	57 (10)
	Rarely	53 (10)
	Never	394 (72)

^a^Percentages may not add to 100 due to missing data

*IQR*, interquartile range; *SD*, standard deviation

### Priorities for living with PMR

Priorities selected as among the five most important aspects of living with PMR can be seen in Table 2. Ninety-seven percent of participants (*n* = 533) indicated at least one priority for living with PMR, with the majority selecting five priorities (although 22 selected more than 5 priorities, 58 selected fewer). Managing stiffness (*n* = 415, 75%) and managing pain (*n* = 406, 74%) were most commonly identified as areas that required support. Ninety percent of participants (*n* = 497) selected at least one of these two options. Other frequently selected priorities were management of steroids and other medications (*n* = 400, 73%), outlook for recovery (*n* = 376, 68%) and things participants could do to help themselves (*n* = 355, 65%). Priorities did not differ by disease duration, using a cutoff of greater or less than 2 years, including stiffness (75% vs 76%), pain (73% vs 76%), steroid management (73% vs 77%), outlook for recovery (72% vs 72%) and things patients could do to help themselves (65% vs 63%).

**Table 2:** Important aspects of living with PMR. Participants were asked to select five priorities

	Selected as a priority, *N* (%)^a^		
Aspect of living with PMR	Males	Females	All
Managing stiffness	140 (75)	274 (76)	415 (75)
Managing pain	130 (70)	275 (76)	406 (74)
Management of steroids and other medications	140 (75)	259 (72)	400 (73)
Outlook for recovery	135 (73)	239 (66)	376 (68)
Things you can do to help yourself	118 (63)	235 (65)	355 (65)
Day to day activities	101 (54)	196 (54)	298 (54)
Non-medical treatments	24 (13)	80 (22)	105 (19)
Developing giant cell arteritis	37 (20)	67 (19)	104 (19)
Work	36 (19)	43 (12)	80 (15)
Contact with other people with the condition	22 (12)	52 (14)	74 (13)

Responses are presented in descending order of frequency

^a^Percentages include participants that did not select any priorities

### Priorities for PMR research

Areas selected as priorities for PMR research, of which any number could be selected, are displayed in Table 3. Ninety-seven percent of participants (*n* = 535) indicated at least one research priority, with a median of 4 priorities (interquartile range 3–5) being selected. Pain, stiffness and fatigue were the research area most commonly prioritised (*n* = 431, 78%), followed by diagnosis (*n* = 393, 71%), steroid management (*n* = 342, 62%) and things patients could do themselves for their condition (*n* = 335, 60%). The risk of developing giant cell arteritis, a key concern for clinicians, was not frequently prioritised by patients and was selected by only 138 (25%). Eight percent of participants (*n* = 42) used the ‘other’ option to describe research priorities beyond those pre-specified. Eight of these responses were inadmissible: one was illegible, two were “do not know” and five had no clear connection to research priorities. Using content analysis, 10 codes were identified (Table 4) from 35 priorities (one participant indicated two distinct priorities). Treatment side effects (*n* = 7), causes of PMR (*n* = 6) and delay in diagnosis (*n* = 6) were the most frequently cited free-text priorities.

**Table 3:** Priorities for PMR research. Participants could select any number of priorities

	Selected as a priority, *N* (%)^a^		
Area of PMR research	Males	Females	All
Pain, stiffness and fatigue	142 (76)	288 (80)	431 (78)
Diagnosis	131 (70)	260 (72)	393 (71)
Steroid management	123 (66)	218 (60)	342 (62)
Things patients with PMR can do for their condition	107 (58)	227 (63)	335 (61)
Multiple health conditions	47 (25)	129 (36)	178 (32)
Alternative and complementary therapies	39 (21)	114 (32)	154 (28)
Developing giant cell arteritis	39 (21)	99 (27)	138 (25)
Role of health professionals	35 (19)	79 (22)	116 (21)
Other (see Table 4)	10 (5)	32 (9)	42 (8)

Responses are presented in descending order of frequency

^a^Percentages include participants that did not select any priorities

**Table 4:** Priorities for PMR research described using the free text ‘other’ option and categorised using content analysis

Area of PMR research	Selected as a priority, *N* (%)^a^
Side effects of treatment	7 (20)
Causes of PMR	6 (17)
Early diagnosis	6 (17)
Self-management of symptoms	5 (14)
Achieving remission	4 (11)
Availability of advice and information	2 (6)
Atypical presentation	2 (6)
Treatment pathways	1 (3)
Effect on ability to work	1 (3)
Testing for GCA by an ophthalmologist	1 (3)

^a^Percentage of admissible responses to the ‘other’ option

## Discussion

PMR is commonly managed in primary care and can have significant long-term impacts for patients. Understanding patient priorities around living with PMR and research priorities is important to ensure research findings generated are relevant to all stakeholders.

This is the first survey of primary care PMR patients to investigate perspectives on the challenges of living with PMR and their priorities for future research. These results highlight that patients are concerned especially with managing symptoms such as pain and stiffness and management of steroids and that these are the areas that patients would prioritise for future research. These findings are similar to previous work surveying PMRGCAuk support group members [4] which highlighted that concerns about steroids are an important issue for patients. Developing giant cell arteritis, a key concern for clinicians [15], was rarely considered as one of the important aspects of living with PMR or as a research priority. It is not clear to what extent this indicates a mismatch between patient and clinical priorities, rather than a lack of patient information regarding giant cell arteritis and its effects.

There are several strengths and weaknesses that need to be considered when interpreting the results of this study. This was a large cohort of PMR patients (550 patients) recruited from across England and as such, the results are likely to be highly generalisable. A limitation is that these patients were included on the basis of a primary care diagnostic code for PMR, rather than having been assessed in specialist services, although the demographics of this population are similar to those seen in both primary [16, 17] and secondary [18] care PMR cohorts. By including patients with a range of disease durations (median 2 years), we may also have captured a different patient experience than those with recent onset disease, although our results suggest that symptom management and medication remains important issues for patients with a longer duration of disease.

In summary, a large primary care survey of people with PMR suggests that management of symptoms such as pain, stiffness and fatigue, diagnosis and managing steroids are key research priorities for patients. Researchers and funding organisations should be aware of these priorities if we are to generate research findings that are relevant to the widest range of stakeholders.
